# 25nd Annual Meeting of the American Society of Breast Surgeons: What Happens in Vegas at ASBrS Does Not Stay in Vegas—Take-Home Messages and Scientific Impact

**DOI:** 10.1245/s10434-025-18136-5

**Published:** 2025-08-28

**Authors:** Kari M. Rosenkranz, Mediget Teshome, Sarah L. Blair

**Affiliations:** 1 Dartmouth Hitchcock Medical Center, Lebanon, NH USA; 2 Department of Surgery, University of California Los Angeles, Los Angeles, CA USA; 3 Department of Surgery, University of California San Diego, San Diego, CA USA

The American Society of Breast Surgeons (ASBrS) convened in Las Vegas, Nevada, April 30 to May 4, 5025, with record attendance of more than 1700 attendees, including 152 international participants from 40 countries. This year the meeting included some always popular courses in addition to some new pre-meeting courses including a leadership course directed by Drs. Chen and Neumayer and a lactation provider course directed by Dr. Katrina Mitchell*.* We had some very engaging questions and discussions in our scientific sessions, demonstrating a very active community. This year we had 426 abstracts submitted. We accepted 18 oral presentations and 8 quick shots in addition to 292 posters.

## General Session Highlights

Given the rapidly changing landscape of breast care, the general session kicked off just past mid-day to increase capacity for educational content including panels, scientific presentations, and six great debates. The perennially well-attended panel on coding and reimbursement easily packed the house despite the early start. The day continued with a multidisciplinary conversation about de-escalation across medical, radiation, and surgical oncology. The panelists offered strategies for working across the specialties to be sure that patients have a voice in when and how to right-size their care given sometimes competing options for de-escalation.

With increasing access to information, many patients are turning to treatments without data provided by clinical trials. A panel on this topic addressed evidence-based complementary approaches to cancer care, the ethics of treating patients who seek treatments “outside the clinical trials,” and the role that religion and spirituality can play in enhancing patient comfort, treatment, and healing.

Day 1 concluded with the first of five debates, which beautifully outlined the pros and cons of ductal carcinoma *in situ* (DCIS) management with observation alone. The debate highlights included presentation of recently published data from the ALLIANCE COMET trial led by Dr. Hwang.

The pace did not let up. Day 2 included two important debates regarding the risk and benefit of contralateral prophylactic mastectomy as well as the role of breast conservation versus mastectomy in an era of improved adjuvant and neoadjuvant non-surgical therapies. Each debate throughout the meeting concluded with an “adjudicator,” a neutral presenter who summarized best practice based on synthesis of the most current evidence. Many sessions also showcased the increasing global impact of the ASBrS, featuring international perspectives and practice patterns.

The day also shone a light on the lesser discussed breast cancer histology-invasive lobular cancer (it is not just ductal). The outstanding panelists reviewed unique features of invasive lobular carcinoma (ILC) including imaging characteristics, genetics, and treatment options. A panel on cultural competencies addressed potential disparities in the care of racially diverse and transgender patients. Amidst all these sessions, keynote speaker, Dr. Bruce Mann, gave a tour de force lecture summarizing much of his own extensive clinical research in his talk entitled “Reimagining Stage 0 and 1 Breast Cancer.”

The highlights of the final 2 days of the meeting included Dr. Helen Pass’s renowned talk summarizing the most impactful papers of the year in breast research as well as the second annual summary of key research from multidisciplinary meetings including the San Antonio Breast Conference, St. Gallen; the American Society of Clinical Oncology (ASCO); the American Society for Therapeutic Radiology and Oncology (ASTRO); and the Lancet Breast Commission. Clinical management in areas of advancement and controversy, most notably when and how to de-escalate axillary surgery, was reviewed via debate and in panel discussion. The Hoag Symposium addressed technical and technologic innovations in breast surgery including the role of robotic mastectomy, lympho-venous bypass and lymph node transfer, extreme oncoplasty, and vacuum-assisted excision.

In a panel focused on shared decision-making, empathy, and effective communication with patients, opera singer Amanda Echalaz, BMus Hon, literally brought down the house presenting her reflections on her experience as a patient and later earned a round of rousing applause for her performance at the ASBrS gala.

And of course, ASBrS President Dr. Judy Boughey’s address motivated all of us not just to engage in clinical trials, but also to dream them. She urged us to boldly ask new questions, look for opportunities in the least expected places, collaborate with colleagues, and design new trials that will challenge the status quo, advance care, decrease morbidity, and extend lives with increased quality for our patients.

After three full days of content, time reconnecting with friends and colleagues, for some, a dance filled gala, and for others, nights enjoying the lights, cuisine, and shows of Vegas, the meeting came to an end. Given the pace of research and change in the care of breast patients, we already look forward to new learning opportunities in Seattle, WA 2026.

## Oral and Scientific Session Highlights

Congratulations to all the participants in our scientific sessions. We had great representation in many areas of breast cancer surgical treatment. This year we had a growing representation from exciting clinical trials demonstrating our organization’s increasing impact in the breast cancer scientific community. For example, we had several papers from the ISPY multicenter trial answering surgical questions such as “Impact of Time to Surgery Post-Neoadjuvant Chemotherapy on Breast Cancer Outcomes: A Retrospective Study of Patients Enrolled in the I-SPY 2 Clinical Trial,” which won the Outstanding Scientific Presentation Award by Dr. Van Hassel. This important study reassures surgeons that we have up to 8 weeks to get patients to the operating room after neoadjuvant systemic therapy without comprising oncologic outcomes. The ISPY trial also was represented by “Disparities in the Surgical Management of the Axilla by Self-Identified Race in the Multicenter Neoadjuvant I-SPY2 Trial” by Dr. Kaur and “Impact of Neoadjuvant Chemotherapy on Surgical Outcomes and Conversion to Node-Negativity in Invasive Lobular Breast Cancer: Analysis of Molecularly High-Risk Tumors by Histologic Subtype on the I-SPY2 Clinical Trial” by Dr. Muktar.

In addition, the ASBrS Scientific Impact Award, which recognizes an outstanding presentation overall in the meeting, was awarded to Mackenzie Joes for her paper entitled, “Pre-Treatment Tumor Collagen Is Associated With Triple-Negative Breast Cancer Pathological Response to Chemo-Immunotherapy.” This study explores mechanisms of chemo/immune therapy resistance by examining differences in collagen in triple-negative breast cancer.

The George Peters award was awarded to Dr. Edwards for her paper, “Survival Benefits and Less Intensive Treatment for Women Diagnosed With Early-Stage Breast Cancer While Participating in Population-Based Screening,” reiterating that screening mammograms do improve survival.

Furthermore, several other interesting clinical trials are included in this issue of *Annals of Surgical Oncology,* such as “Ninety Days of Preoperative Endocrine Therapy Informs Patient and Physician Preference for Radiation Therapy: Primary Results From the Preoperative Window of Endocrine Therapy to Inform Radiation Therapy Decisions (POWER) Trial” by Dr. Showalter. This paper presents the phase 2 results. Dr. Showalter’s Alliance trial is accruing patients, and ASBrS members are encouraged to participate in this trial involving study to make sure that our elderly patients get appropriate care even if they do not tolerate endocrine therapy. Additionally included in this issue is the “Impact of Quality Improvement Interventions on Biopsy to Treatment Time in Breast Cancer: Results From the PROMPT Quality Collaborative of the National Accreditation Program for Breast Centers” by Dr. Yao’s group. This is a multi-center study from the National Accreditation Program for Breast Center, giving us a road map to improve time from biopsy to treatment.

Additional studies in this issue include a paper from Dr. Cortina’s group entitled “From Podium to PubMed: Successful Manuscript Publication of Oral Breast Surgery Abstract Presentations at National Meetings from 2017 to 2022.” This paper shows that ASBrS authors are more successful publishing their work in journals in part due to extra resources provided by our organization, including support from the Annual Meeting Scientific Committee. Another fun paper is entitled “Is This the Right Link? How TikTok Views Oncoplastic Breast Surgery” by Boolbol’s group, demonstrating that our patients may be receiving incorrect information from patient-created videos, but valuable information from surgeon-created videos. There is more room for ASBrS TikTokers to upload videos with good content. Finally, although it reports a retrospective study, the paper entitled “Added Value of Fine-Needle Aspiration (FNA) or Breast Magnetic Resonance Imaging (MRI) as Part of the ‘Triple Test’ to Assess Palpable Abnormalities on Breast Exam With Negative Imaging Findings” by Dr. Marti showed that we may be on the way to retiring the triple test. Mammography’s negative predictive value is improving, and we may be able to assure patients with palpable masses and a negative mammogram that active surveillance is enough.

Several papers discuss the benefit of neoadjuvant chemotherapy for metaplastic breast cancer tumors, all showing that these tumors do not respond to conventional chemotherapy. Each tumor has a slightly different spin, but overall, everyone concluded that better systemic treatments are needed for this rare but aggressive type of breast cancer. Hopefully newer treatments such as immunotherapy will improve outcomes.

Finally, we thank the members of the Annual Scientific Meeting Committee for reviewing abstracts and diligently reviewing the ensuing manuscripts to give us what we believe is an excellent October issue of *Annals of Surgical Oncology*. We have a very engaged committee membership that continues to move our field forward and strives to select the best papers and educate our members on most cutting-edge topics in breast surgery. The ASBrS continues to impact breast cancer research as it disseminates our findings world-wide. Results of ASBrS data do not stay in Vegas.

